# Lead Extraction of Cardiac Rhythm Devices: A Report of a Single-Center Experience

**DOI:** 10.3389/fcvm.2017.00018

**Published:** 2017-04-13

**Authors:** Ana Isabel Azevedo, João Primo, Helena Gonçalves, Marco Oliveira, Luís Adão, Elisabeth Santos, José Ribeiro, Marlene Fonseca, Adelaide V. Dias, Luís Vouga, Vasco Gama Ribeiro

**Affiliations:** ^1^Cardiology, Vila Nova de Gaia/Espinho Hospital Centre, Vila Nova de Gaia, Portugal; ^2^Cardiothoracic Surgery, Vila Nova de Gaia/Espinho Hospital Centre, Vila Nova de Gaia, Portugal

**Keywords:** lead extraction, lead removal, cardiac implantable electronic device, infection, risk factors

## Abstract

**Introduction and objectives:**

The rate of implanted cardiac electronic devices is increasing as is the need to manage long-term complications. Lead removal is becoming an effective approach to treat such complications. We present our experience in lead removal using different approaches, analyzing the predictors of the use of mechanical extractors/surgical removal.

**Methods:**

Retrospective analysis of lead extractions in a series of 76 consecutive patients (mean age 70.4 ± 13.8 years, 73.7% men) between January 2009 and November 2015.

**Results:**

One hundred thirty-five leads from permanent pacemakers (single chamber 19.7%; dual-chamber 61.8%), implantable cardioverter defibrillators (5.3%), and cardiac resynchronization devices (CRT-P 2.6%; CRT-D 7.9%) were removed, 72.5 ± 73.2 months after implantation. A total of 45.9% were ventricular leads, 40.0% atrial leads, 8.9% defibrillator leads, and 5.2% leads in the coronary sinus; 64.4% had passive fixation. The most common indications for removal were pocket infection (77.8%), infective endocarditis (9.6%), and lead dislodgement (3.7%). A total of 76.3% of the leads were explanted, 20.0% were extracted, and 3.7% were surgically removed. Extraction of the entire lead was achieved in 96.3% of the procedures. After logistic regression (age adjusted), time since implantation was the sole predictor of the need of mechanical extractors/surgical removal. All patients were discharged without major complications. There were no deaths at 30 days.

**Conclusion:**

Our experience in lead removal was effective and safe. Performing these procedures by experienced electrophysiologists with an adequate cardiothoracic surgery team on standby to cope with any complications is required. Referral of high-risk patients to a high-volume center is recommended to optimize clinical success and minimize procedural complications.

## Introduction

Over the past decades, the use of cardiac implantable electronic devices (CIED) has increased significantly. Along with patients’ longer life expectancy, this has led to an increase in the number of device-related complications and, consequently, the growing need to perform lead removal (1, 2). Lead removal is nowadays a specialized procedure, with well-defined indications (3). Several studies from high-volume centers have demonstrated that it can be performed with high success and low complication rates, by employing various methods (4). As this procedure is becoming increasingly frequent in daily practice, reporting objective data is the cornerstone to accurate risk assessment, ultimately improving patients’ outcomes. Few centers in Europe have historically reported on these procedures. In 2012, a document was published reflecting on the current practice on lead removal among 164 centers in 30 European countries, with results and complication rates similar to main international registries (2). More recently, a large prospective, multicentre, European controlled registry of consecutive patients undergoing total lead extraction procedures in European countries, including a follow-up phase, has been performed (ELECTRa). The primary objective was to evaluate the acute and long-term safety of these procedures and included more than 3,500 patients from more than 100 centers, reflecting the present importance of this issue (5).

The aim of this retrospective single-center study is to present our experience in lead removal, using different techniques, analyzing the predictors of the use of mechanical extractors or open-chest lead extraction. Through the evaluation of indications for lead removal, short-term safety, and major procedure-related complications, this study will comprise valuable information for future comparison of data with other centers and countries, ultimately aiming to increase the quality of care in the lead extraction process.

## Materials and Methods

### Study Population

The study included consecutive patients referred to lead removal at a non-university tertiary care center over a 70-month period (from January 2009 to November 2015). All patients gave their informed and written consent to undergo the interventions.

### Data Collection and Indications

Collected data regarding patients’ clinical characteristics, implanted devices and leads, and the procedure were entered into a computerized database and retrospectively analyzed.

### Definitions and Endpoints

The Heart Rhythm Society Expert Consensus recommendations (3) on definition of lead explant/extraction were adhered to. Lead explant was defined as the removal of the lead using simple traction techniques, without using any additional tool. Lead extraction was defined as the removal of the lead, regardless of duration of implant, using specialized equipment including mechanical sheaths, with or without locking stylets. Open-chest lead extraction was defined as the removal of the lead through a sternotomy or thoracotomy.

The leads were separated from the scar tissue by dissection. If manual traction was not successful, a systematic approach using locking stylets (Liberator^®^ Locking Stylet, Cook Medical, USA) and mechanical dilation polypropylene sheaths was used. When necessary, the Evolution^®^ Controlled-Rotation Dilatation Sheath (Cook Medical, USA), consisting of a flexible sheath with a distal threaded metal tip, in which a handle is attached to the plastic sheath proximally and rotates it, allowing the threaded metal end to run through adhesions, was used. Laser-assisted lead extraction was not used.

We defined the endpoints based on the intention-to-treat analysis. Clinical success was achieved whenever the entire lead was completely removed. Partial success was noted when most of the lead was removed, but not completely (leaving part of the coil and/or the tip). Failure was defined when both previous endpoints were not fulfilled.

### Statistical Analysis

Continuous variables are presented as mean and SD, and categorical variables are presented as frequency and percentage. Logistic regression was used to analyze the predictors of mechanical extraction or surgical removal. SPSS software (version 22.0, SPSS, Inc., Chicago, IL, USA) was used.

## Results

A series of 76 consecutive patients (mean age 70.4 ± 13.8 years, 73.7% men) underwent removal procedures of a total of 135 leads. Fifty-five (40.7%) of the leads were removed from patients referred from other centers. Sixty-three patients (81.5%) underwent removal of permanent pacemaker leads. Right ventricle pacing leads accounted for the majority of the leads removed, followed by atrial leads, defibrillator leads, and coronary sinus leads. Lead tip fixation was passive in 87 leads (64.4%) and active in 17 leads (12.6%). Mean time since implantation was 72.5 months (range 1–252 months). Table 1 shows the study population characteristics, type of device implanted, location, and fixation mechanism of the leads.

**Table 1:** **Patient and lead characteristics**.

Men	56 (73.7)
Age (years)	70.7 ± 13.8
Time after implantation (months)	72.5 ± 73.2
Implanted device	
Single-chamber pacemaker	15 (19.7)
Dual-chamber pacemaker	47 (61.8)
Biventricular pacemaker (CRT-P)	2 (2.6)
Single-chamber implantable cardioverter-defibrillator (ICD)	4 (5.3)
Biventricular ICD (CRT-D)	6 (7.9)
Unknown	2 (2.6)
Leads	
Pacing leads	
*Right atrium*	54 (40.0)
*Right ventricle*	62 (45.9)
*Left ventricle (coronary sinus)*	7 (5.2)
Defibrillator leads	12 (8.9)
Fixation method	
Active	17 (12.6)
Passive	87 (64.4)
Unknown	31 (23.0)

*Data are presented as number (percentage) or mean ± SD*.

Indications for the procedure were pocket infection (105 leads, 77.8%), infective endocarditis (13 leads, 9.6%), lead dislocation (5 leads, 5.7%), skin erosion (4 leads, 3.0%), and other indications (8 leads, 5.9%). The indications for lead removal are displayed in Table 2.

**Table 2:** **Indication for lead removal**.

Pocket infection	105 (77.8)
Infective endocarditis	13 (9.6)
Lead dislocation	5 (5.7)
Skin erosion	4 (3.0)
Insulation defect	3 (2.2)
Myocardial perforation	2 (1.5)
Pectoral stimulation	2 (1.5)
Device upgrade	1 (0.7)

*Data are presented as number (percentage)*.

There was a median of two leads removed per patient (range 1–3). Fifty-nine patients underwent lead explant, 14 underwent extraction, and 3 patients were submitted to open chest extraction. Of the 135 leads removed, 103 (76.3%) were explanted, 27 (20.0%) were extracted, and 5 (3.7%) removed through open chest lead extraction. The surgically extracted five leads were removed from three patients: one patient had infective endocarditis of a dual-chamber pacemaker, with a large and highly mobile vegetation on the ventricular lead, and was submitted to surgical extraction of both leads, followed by implantation of an epicardial lead; another patient had a defibrillator lead removed due to right ventricle perforation; and one patient was submitted to surgical removal of auricular and ventricular leads, as well as valvular aortic mechanical prosthesis substitution due to infective endocarditis of the prosthesis, with implantation of an epicardial lead. There was a high risk in percutaneous removal of the leads in these patients, and they were directly referred to surgical extraction.

Clinical success was achieved in 130 (96.3%) of the leads and partial in 4 leads (3.0%). Of the four leads incompletely removed, only small fragments were left in place, and they corresponded to two explants of ventricular leads of dual-chamber pacemakers, one explant of an auricular lead of a dual-chamber pacemaker, and an extraction of a ventricular lead of a dual-chamber pacemaker. On one of the lead extractions, no registries were available.

After logistic regression (age adjusted), time since implantation, but not the type of lead (pacing versus defibrillator lead) or the type of fixation (passive versus active), was a predictor of the need to use mechanical extractors or surgical removal of the lead (OR 1.03; CI 95% 1.01–1.05, *p* = 0.001).

All patients were discharged without major complications, and there were no deaths at 30 days.

## Discussion

The present article reports a single-center experience in lead removal using simple traction techniques or mechanical/surgical extraction. We were able to completely remove leads in 96.3% of the patients, results that are consistent with several publications (1, 4, 6–8). These data support previously published evidence that the operators’ experience and the volume of procedures are critical factors for success rate of the procedures (6–8). In recent years, lead removal has becoming increasingly common worldwide as a consequence of the growing prevalence of patients using pacemakers, implantable cardioverter defibrillators, and cardiac resynchronization devices who are at risk of device complications requiring removal of the implanted system (1, 7). In our study, lead explant, using simple manual traction, was successfully used in the majority of the procedures (76.3% of the leads removed). Applying traction force to the proximal end of the lead has been the earliest and simplest approach to lead removal, especially in recently implanted ones. As time since implantation increases, fibrosis around the lead body and between leads develops and simple traction may not suffice (8). In the past decades, significant progress has been made in lead extraction techniques: instead of continuous traction, new countertraction techniques have been developed, which is composed of mechanical systems for the removal of fibrous adhesions in the venous system (1). In our series, longer time since implantation determined the need to use a mechanical approach or surgical extraction of the leads. In 20.0% of the leads, we used mechanical lead extraction devices, with or without locking stylets (Cook Medical, USA). In 3.7% of the leads, a surgical removal had to be performed due to the high risk of the procedures. A series of extraction techniques using application of laser, radiofrequency energy, or other cutting technologies have been purposed as more effective than mechanical-only extractions. However, reports have shown that laser extraction sheaths are not associated with a higher rate of complete extraction compared to conventional methods and are one of the predictors of major complications (8). Furthermore, these techniques are not available in the majority of the centers. As so, the use of different approaches depends on patient’s clinical presentation, operator’s skills and experience, and locally available material (1). Considering that the method of lead explant was predominant compared to the other extraction modalities, we can speculate that our population was overall healthier compared with other international registries, which could contribute to the high success rate and low complication rate achieved.

The most common reason for lead removal in our patients was infection. In fact, CIED-associated infections are the strongest indication for complete CIED system removal. However, clinical scenarios of CIED infections are broad: patients can present with nothing more than pain in the pocket to obvious signs of infection, namely fever, bacteremia, vegetations, and sepsis. When an infection is identified, there is a strong indication for removal of all components of the CIED system, including the device, leads, and caps. When blood cultures obtained in different days are consistently positive, even when there is no clear source, transvenous lead removal should be strongly considered. Nevertheless, even in patients with documented device-related infection, blood cultures can be negative, even in the absence of antibiotic therapy. Considering antibiotic treatment, no clinical trials have tested the minimal duration of antibiotic treatment or the optimal time to switch from intravenous to oral therapy, but there is a robust experience in applying the non-CIED-related endocarditis guidelines (3).

In our study, there were no major complications related to the procedure or deaths at 30 days. Although lead removal has developed to be an effective and safe procedure, complications, occasionally life-threatening ones, are reported to occur. In previously published data, procedural mortality rates range from 0.1 to 0.6% and major complication rates from 1.4 to 1.9% (9). A recently published study analyzing risk factors for short- and long-term mortality after ICED infection concluded that heart failure, chronic corticosteroid therapy, and presentation with ICED-related infective endocarditis were associated with both 30-day and long-term mortality (10). In fact, in the majority of cases, death can occur not directly related to the extraction procedure or its complications, but rather the need for extraction represents a marker of the severity of the infectious process and patients’ comorbidities, which increase their mortality risk (1). In our series, identification of risk factors for short- and long-term mortality could not be performed.

### Study Limitations

This study is a retrospective analysis and is subject to the inherent limitations of such studies. These results should be interpreted in the light of a single-center experience and cannot be generalized. The number of patients is relatively small, and only the patients treated and leads consecutively extracted were analyzed. Therefore, two kinds of selection bias may have occurred: on the one hand, the non-referral of patients for lead removal in our center for various reasons; on the other hand, a number of patients who did not underwent lead removal due to high procedural risk. Another limitation is the lack of events at 30-day follow-up and absence of long-term follow-up, which made the identification of predictors of complications and mortality impossible.

## Conclusion

Our experience in removing leads from CIED has proven to be effective and safe.

Performing these procedures by experienced electrophysiologists with an adequate cardiothoracic surgery team on standby to cope with any complications is required.

Referral of high-risk patients to higher volume centers is recommended to optimize clinical success and minimize procedural complications.

## Informed Consent

This study was carried out in accordance to the recommendations of the Heart Rhythm Society on transvenous lead extraction. Written informed consent was obtained from all patients prior to the interventions. The authors declare that no experiments were performed on humans, and no patient data are showed in this article.

## Ethics Statement

This study was carried out in accordance with the recommendations on cardiac rhythm device extraction. All subjects gave written informed consent in accordance with the Declaration of Helsinki.

## Author Contributions

All of the authors gave substantial contribution to the conception and design of the work and acquisition, analysis and/or interpretation of the data. All of the authors provided critical revision for important intellectual content and gave their final approval of the version to be published.

## Conflict of Interest Statement

The authors declare that the research was conducted in the absence of any commercial or financial relationships that could be construed as a potential conflict of interest.
